# Pregnancy Toxemia in Ewes: A Review of Molecular Metabolic Mechanisms and Management Strategies

**DOI:** 10.3390/metabo13020149

**Published:** 2023-01-18

**Authors:** Xiaoyu Ji, Ning Liu, Yuqin Wang, Ke Ding, Shucheng Huang, Cai Zhang

**Affiliations:** 1 College of Animal Science and Technology, Henan University of Science and Technology, Luoyang 471023, China; 2 College of Veterinary Medicine, Henan Agricultural University, Zhengzhou 450002, China

**Keywords:** pregnancy toxemia, nutritional metabolic disease, lipid metabolism, diagnosis, small ruminants

## Abstract

Pregnancy toxemia is a nutritional metabolic disease during late gestation in small ruminants. The condition is characterized by disorders in carbohydrate and fat metabolism. Obese and multiparous ewes are particularly susceptible to pregnancy toxemia, which may lead to maternal death, abortion, or premature birth. Highly productive multiparous meat ewes are major breeding animals, which has led to an increased incidence of the disease. However, the pathogenesis of pregnancy toxemia remains unclear and adequate disease prevention and treatment strategies are absent. Investigating the pathogenesis of pregnancy toxemia, especially the metabolic pathways of hepatic lipids, is key to an improved understanding of the condition. This review provides a snapshot of the genes that are associated with lipid metabolism in the ovine liver, including genes involved in fatty acid oxidation, acetyl coenzyme metabolism, and triglyceride synthesis; describes the interrelationships between these genes; and summarizes the diagnosis, prevention, and treatment of pregnancy toxemia.

## 1. Introduction

Pregnancy toxemia at the end of gestation in ewes is a nutritional metabolic disease that arises because of impaired carbohydrate and fatty acid metabolism. Nutritional deficiency in ewes during late pregnancy is the main risk factor for the condition, which is caused by insufficient energy in the diet or by a decrease in rumen capacity due to fetal growth. The incidence of the disease is approximately 5% to 20%, with mortality rates up to 80% in untreated animals. Moreover, 40% of ewes die despite intensive treatment strategies [1]. In addition, the disease may lead to abortion and stillbirth, and also may cause acute toxemia and death in ewes when the fetus dies in the mother [2].

Pregnancy is an exceptional period during which ewes must consume sufficient nutrition to maintain maternal body metabolism, as well as to guarantee adequate fetal growth. Fat accumulation ensures that ewes obtain sufficient energy to sustain fetal development and to meet lactation requirements. However, ewe appetite is suppressed during pregnancy and the animals remain in a negative energy balance for a lengthy period, which may inhibit basic cellular processes, including DNA replication and cell cycle progression, and thereby affect the growth and development of the fetal liver [3]. Mobilization of excess fat in this situation may lead to lipid accumulation in the mother’s liver and fetal liver. Pregnant ewes are highly sensitive to environmental changes. Abrupt alterations in feeding environment and management induce intestinal microbiome changes and stress and make the animals more susceptible to disease. Undernutrition changes the colonic bacterial diversity and composition in pregnant ewes, including an increased abundance of unclassified Peptococcaceae and decreased abundances of Ruminococcus, unclassified Ruminococcaceae, and unclassified VadinBB60 [4]. Rumen epithelium barrier function was compromised by severe feed restriction, as it decreased most ruminal fermentation parameters and altered the composition of rumen epithelium-associated microbes [5]. In parallel, acidosis, dehydration, hypocalcemia, parasitic infections, or chronic wasting diseases may lead to secondary pregnancy toxemia [6]. In addition, lean ewes are more susceptible to starvation pregnancy toxemia due to malnutrition [7]. Moreover, multiparous ewes may have increased difficulty in producing glucose and clearing ketone bodies, which also make the animals more susceptible to pregnancy toxemia [8].

Pregnancy toxemia often is accompanied by hypoglycemia, hyperglycemia, lipemia, and ketosis [1,9]. Increased levels of ketone bodies due to the disease may cause dehydration and electrolyte metabolism disorders [10]. Diseased ewes display diverse symptoms, including decreased appetite, depression, staggering gait, rough skin, blindness, muscle tremors, grinding teeth, convulsions, and eventually coma and death [11,12]. Monitoring of hematological indicators, serum biochemical indicators, and ketone body levels in ewes allow for early detection and treatment of pregnancy toxemia.

The authors searched the National Center for Biotechnology Information and China National Knowledge Infrastructure for publications on gestational toxemia in sheep and goats; ketosis in dairy cows; nutrient digestion and metabolism in ruminants; hepatic fat metabolism pathways and their molecular mechanisms; multiple birth genes in sheep; and diagnosis, treatment, and prevention of nutritional metabolic diseases. The authors identified certain points, including molecular regulatory mechanisms that underpin pregnancy toxemia, which are unclear, especially with respect to disorders of liver lipid metabolism. Here, we review the genes that may be implicated in the disease and analyze the interconnections between these genes. These insights are especially pertinent for future studies that aim to understand the condition and are geared at designing effective prevention and treatment strategies.

## 2. Glycolipid Metabolism in Ruminants

### 2.1. Glucose Metabolism in Ruminants

**Figure 1:**
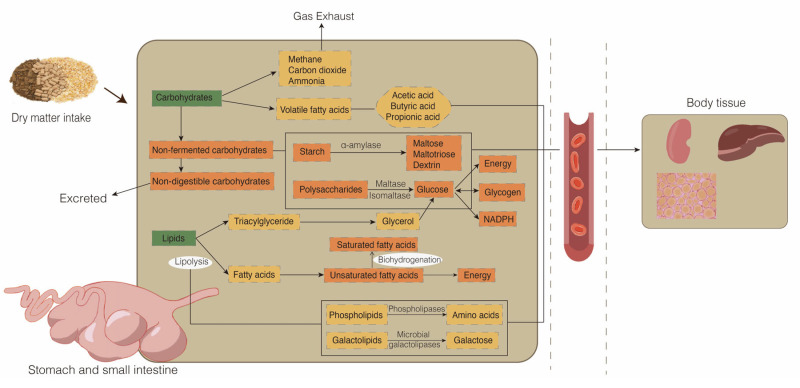
Metabolic processes of glucose and lipid metabolism in ruminants. Green indicates nutrient intake from dry matter; yellow indicates nutrient metabolism in the rumen; orange indicates nutrient metabolism in the small intestine.

The supply of nutrients in animals is key to maintaining stable growth metabolism and production performance. Carbohydrates are indispensable for these processes. Most carbohydrates, particularly cellulose and starch, are not available directly in the diet of ruminants. Instead, carbohydrates are digested and absorbed mainly by two methods (Figure 1). First, the major products of the rumen microbial fermentation of carbohydrates are volatile fatty acids (mainly comprising acetic acid, propionic acid, and butyric acid), methane, carbon dioxide, and ammonia. Second, the hydrolysis of nonfermentation carbohydrates in the small intestine produces glucose, which provides energy, glycogen, and NADPH. The first stage of nonfermentation carbohydrate hydrolysis begins with the secretion of duodenal pancreatic α-amylase, which hydrolyzes starch to release maltose, maltotriose, and dextrin. Subsequently, small polysaccharides are hydrolyzed into individual glucose moieties by maltase and isomaltase enzymes that are secreted in the epithelial brush border membrane of the small intestine. Finally, glucose in the intestinal lumen of the small intestine is transported into the portal circulation system [13,14,15]. In addition, gluconeogenesis in the liver produces large amounts of glucose for energy supply. During prolonged starvation or ingestion of low-carbohydrate diets, glucose production from gluconeogenesis may account for 90% of the circulating glucose [16].

### 2.2. Lipid Metabolism in Ruminants

Dietary lipids are metabolized in the rumen through biohydrogenation and lipolysis (Figure 1). Most of the lipids in the feed are hydrolyzed completely by rumen microorganisms to produce glycerol and fatty acids. In this process, lipases, phospholipases, and microbial galactolipases hydrolyze the ester bonds of complex lipids, including triacylglycerides (TG), phospholipids (PL), and galactolipids, leading mainly to non-esterified fatty acids and glycerol, in addition to the amino acids derived from PL and galactose derived from galactolipids [17]. Hydrogenation of lipids is the conversion of unsaturated fatty acids, which either enter the rumen or are derived from triglycerides, to saturated fatty acids by the action of microorganisms. Saturated fatty acids, biohydrogenation intermediates, and microbial PL are principally available for absorption in the small intestine after ruminal biohydrogenation [18,19]. The synthesis of TG, PL, cholesterol, and apoproteins produces chylomicrons, which are secreted to the lymph and then taken up by the blood through the thoracic duct [20].

## 3. Causes of Pregnancy Toxemia

**Figure 2:**
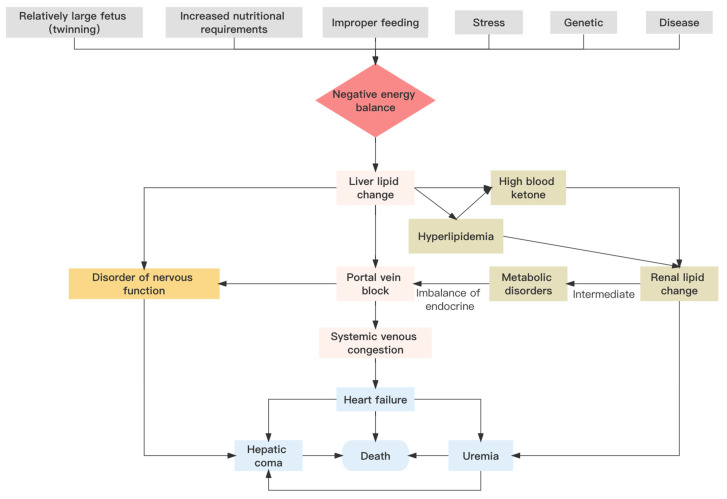
Pathogenesis of pregnancy toxemia in sheep.

Pregnancy toxemia in ewes involves a spectrum of symptoms that may be generated by numerous different causative agents. However, the main trigger of the disease is a negative energy balance [21,22]. Specifically, pregnancy toxemia in sheep is due to an imbalance in glucose metabolism, which leads to disorders in the turnover of carbohydrates, lipids, proteins, and other nutrients, and results in hypoglycemia and disease [23]. Nutritional, genetic, physiological, environmental, and other factors individually or synergistically may influence the incidence of pregnancy toxemia (Figure 2).

### 3.1. Nutrition and Digestion

Nutritional support is essential during gestation to maintain a healthy pregnancy. In late pregnancy, the energy requirement of ewes carrying twins and triplets increases by 180% and 240%, respectively [7]. Similarly, maternal and fetal health depends on obtaining sufficient vitamins and minerals. A failure to meet these nutritional requirements may predispose to pregnancy toxemia. The continued growth of the fetus causes a reduction in rumen capacity, which may result in insufficient maternal nutrient intake. Obese ewes are more prone to the condition: fat occupies a significant proportion of the ewe body, which reduces the capacity of the rumen to accommodate feed and achieve sufficient digestion [23]. The maternal body through the endocrine system adapts the metabolism of fats, carbohydrates, and proteins to provide nutrients for fetal growth and development [24]. Metabolic homeostasis may be disrupted by intermittent changes in metabolic status. However, as the supply of nutrients continues to decrease, this metabolic process leads to elevated production of ketone bodies [25]. The ewe ultimately is unable to clear ketone bodies in time, and pregnancy toxemia occurs [1,26]. Ewes may be challenged by weather, sudden feed changes, or other environmental alterations, which may lead to changes in the rumenal and intestinal microbiome including a stress reaction that perturbs metabolism. The sheep must mobilize large amounts of energy to deal with these stressors, which result in enhanced catabolism and reduced anabolism of nutrients. In addition, stress reactions lead to decreased immunity and induce disease, which also contributes to the development of pregnancy toxemia [27].

### 3.2. Genetics

Genetic factors, which have a higher incidence in sheep than in goats [28], may be significant in pregnancy toxemia. For example, multiparous ewes are prone to a negative energy balance during pregnancy, which results in lipid metabolism disturbances and pregnancy toxemia due to competition for nutrients between mother and baby [29]. These observations suggest that genetic factors may exert a key role in the development of the multiparous condition. Genes that encode fecundity booroola (*FecB*), bone morphogenetic protein receptor type 1B (*BMPR-IB*), bone morphogenetic protein 15 (*BMP15*), and growth differentiation factor 9 (*GDF9*); all of these genes are mutated to varying degrees, controlling reproductive behavior in sheep. These and other genes increase ovulation and lambing rates [30,31,32,33], affect ovarian development [34], and regulate gonadotropin secretion [35]. The *FecB* gene is a major gene associated with sheep prolificacy, and is a potential candidate gene for genetic control of sheep reproductive performance [36]. Ewes with the type BB (mutant pure) gene of *FecB* are more susceptible to pregnancy toxemia in sheep, and it has also been demonstrated that ewes with multiple pregnancies are more susceptible to pregnancy toxemia [37]. Bone morphogenetic proteins, as members of the transforming growth factor-β (TGF-β) family, regulate the granulosa cell processes, which lead to the development of ovarian follicles [38]. Bone morphogenetic protein 15 and GDF9 within the follicle regulate cell proliferation and differentiation by intercellular signaling between the oocyte and granulosa cells [39]. Knockout of the *BMP15* gene leads to reduced fertility and lower ovulation and fertilization rates in female mice [40]. As more multiparous genes are discovered and selected for, consideration needs to be given to strategies that keep ewes free of pregnancy toxemia while retaining the canonical function of these genes.

## 4. Lipid Metabolism-Related Molecular Mechanisms in Pregnancy Toxemia

**Figure 3:**
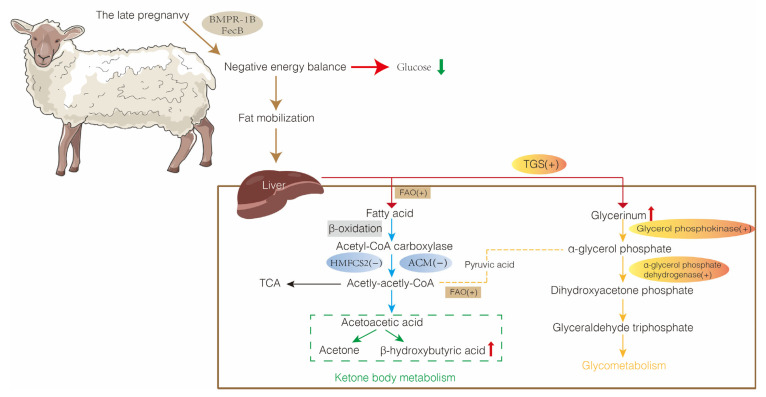
Metabolic mechanisms of pregnancy toxemia in the liver of sheep. TCA, tricarboxylic acid; FAO, fatty acid oxidation; ACM, acetyl coenzyme metabolism; TGS, triglyceride synthesis; HMGCS2, hydroxy methylglutaryl-CoA synthase 2.

The liver is a vital metabolic organ that is involved in all aspects of lipid metabolism (Figure 3). Ewes with pregnancy toxemia typically are in a negative energy balance that affects fatty acid oxidation (FAO), acetyl coenzyme metabolism (ACM), and triglyceride synthesis (TGS), which results in disturbed lipid metabolism and impaired liver function. Investigating the molecular mechanisms that direct lipid metabolism in the ewe liver is essential for the prevention and treatment of pregnancy toxemia.

### 4.1. Fatty Acid Oxidation-Related Genes

Fatty acid oxidation entails the hydrolysis of lipids to produce glycerol and fatty acids with the release of large amounts of energy (Table 1). The process involves the sequential steps of fatty acid activation, lipid acyl CoA transfer, β-oxidation of lipid acyl CoA, and thiolysis. The liver and muscle are the most active tissues for fatty acid oxidation, and β-oxidation is a key process in fatty acid metabolism (Figure 3).

Fat tissue in ewes that are in negative energy balance produces increased amounts of non-esterified fatty acids (NEFAs) to provide energy [41]. The elevated concentration of NEFAs results in increased expression of long chain acyl-CoA synthetase (ACSL), long chain acyl-CoA dehydrogenase (ACADL), and carnitine palmitoyltransferase I (CPT1) enzymes that are involved in fatty acid metabolism [42,43]. Non-esterified fatty acids accumulate in the liver and blood, and expression of FAO-related genes (*ASCL*, *CPT1*, *ACADL*, hydroxyacyl-CoA dehydrogenase [*HADHA*], enoyl-CoA hydratase [*ECH*], and acetyl-Coenzyme A acyltransferase [*ACAA*]) increases during ovine pregnancy toxemia [20]. In addition, the expression of the *ACSL*, *ACDL*, and *CPT1* genes was enhanced significantly with an increase in NEFAs in cultured bovine hepatocytes, which leads to hepatic lipid metabolism disturbance and hepatic steatosis [42,44]. Ewes and cows exhibit some similarities with respect to the effects of lipid metabolism disorders, including ketosis in cows. We speculate that lipid metabolism disorders may be ameliorated by regulating the expression of these genes.

The *ACSL1* gene, a member of the *ACSL* family, is expressed primarily in the liver and is a target gene for peroxisome proliferator-activated receptor alpha (PPARα) [45]. PPARα is a major regulator of hepatic free fatty acid metabolism, with roles in fatty acid uptake, esterification, and transport, as well as regulation of lipoprotein and cholesterol metabolism, adipogenesis, and ketogenesis [46,47,48]. Activation of the PPARα/RXRA signal pathway improves FAO and ketogenesis while reducing the accumulation and esterification of NEFAs [41]. Furthermore, *ASCL1* overexpression reduces fatty acid oxidation in the peroxisome proliferator-activated receptor γ (PPARγ) pathway, which leads to the accumulation of TGs and excessive lipid deposition in the liver [49]. Carnitine palmitoyltransferase I is thought to be the rate-limiting enzyme of FAO and mediates the transport of ACSL [50]. In addition, TANK-binding kinase 1 regulates the localization of ACSL1 and modulates fatty acid oxidation [51].

### 4.2. Acetyl Coenzyme Metabolism-Related Genes

Acetyl coenzyme A (Acetyl CoA) is a product of pyruvate oxidative decarboxylation and fatty acid β-oxidation (Figure 3). Acetyl coenzyme A is a metabolic intermediate and second messenger, and influences numerous important hepatocyte processes, including fatty acid synthesis, cholesterol production, and ketone body synthesis [52]. Acetyl coenzyme A is converted to ketone bodies, mainly acetoacetic acid (AcAc) and β-hydroxybutyrate (BHBA), by ketogenesis in the liver mitochondrial matrix [53,54]. Glucose, lipid, and protein enter the tricarboxylic acid cycle (TCA) and oxidative phosphorylation via acetyl CoA, which is completely oxidized to produce carbon dioxide and water, releasing energy for ATP synthesis. Nutritional restriction reduces glucose levels and glycolytic metabolism, which induces lipolysis and ketogenesis in adipose tissue, as well as increases the mitochondrial oxidation of non-glucose energy substrates into acetyl CoA, which stimulates the TCA cycle for energy production [55,56]. Pregnancy toxemia in ewes inhibits expression of the ACM-related genes 3-hydroxy-3-methyl-glutaryl-coenzyme A (*HMG-CoA*), 3-hydroxy-3-methylglutaryl coenzyme A synthase 1 and 2 (*HMGCS1* and *HMGCS2*), and 3-hydroxy-3-methylglutaryl-CoA reductase (*HMGCR*) [20]. The inhibition of the ACM pathway causes an inability to provide energy by the usual metabolic route, and therefore NEFAs will continue to be produced as a substitute energy source. This perturbation causes the accumulation of NEFAs and the formation of fatty liver. Non-esterified fatty acid concentrations in prenuptial sheep decline over time, whereas NEFAs increase during pregnancy in mature sheep and then decline after lambing, which marks the end of negative energy balance and fat mobilization [57]. Increased NEFA concentrations and BHBA levels and decreased *HMGCS* gene expression levels were observed in ketotic cows after fasting [58]. High concentrations of BHBA may induce oxidative stress in calf hepatocytes and affect the function of the liver in cows [59]. Moreover, restricted feeding inhibits the expression of HMGCS enzymes and HMG-CoA in the liver of ketosis cows [60].

Hydroxy methylglutaryl-CoA synthase 2 catalyzes the production of HMG-CoA and free CoA from acetoacetate coenzyme A (AcAc-CoA) and acetyl CoA [53,61] (Table 1). *Hydroxy methylglutaryl-CoA synthase 2* is a rate-limiting enzyme for the synthesis of BHBA and exerts a prominent role in regulating ketogenesis [62]. The β-hydroxybutyrate activates Adenosine 5‘-monophosphate (AMP)-activated protein kinase (AMPK), which increases glucose uptake in skeletal muscle, promotes insulin sensitivity and fatty acid oxidation, and regulates the transcription of certain genes [63,64]. Adenosine 5‘-monophosphate-activated protein kinase is a major regulator of glucose and lipid metabolism and reduces lipid production in the liver by decreasing the expression of *HMCGS* and *HMCGR* [65]. Non-esterified fatty acids inhibited AMPK activity, increased the expression of genes involved in lipid synthesis and glycolysis, and decreased the expression of genes required for glycogen synthesis in a hepatocyte model of laying hens treated with NEFAs [66]. Moreover, activation of AMPK inhibited hepatocyte proliferation and reduced steatosis and liver injury in an obese murine model [67]. These observations suggest that investigation of the significance of AMPK as an activator of lipid-associated genes in ovine pregnancy toxemia will provide important clues concerning the mechanism of disease formation and progression.

The inhibition of ACM in ewes may be related to modulation of expression of FAO genes: PPARγ decreased the expression of *HMGCS2* and the production of BHBA [62]. Continuous production and accumulation of BHBA, as well as feedback regulation of the ketone body production pathway, resulted in inhibiting ACM and exasperating fatty liver. However, no solution to the problem of ovine pregnancy toxemia has been proposed based on these findings.

### 4.3. Triglyceride Synthesis and Related Genes

Triglycerides are an efficient energy source. The main routes of TG formation in hepatocytes involve the esterification of NEFAs to produce TGs, which are stored in hepatocytes in the form of lipid droplets, and glycolipid conversion, in which fatty acids are synthesized from acetyl-CoA and esterified to produce TGs. Triglycerides produced in the liver are transported by very low-density lipoproteins to adipose tissue for storage.

The expression of the TGS-related genes for glycerol kinase (GK), glycerol-3-phosphate dehydrogenase (GPD), glycerol-3-phosphate acyltransferase (GPA), and diacylglycerol acyltransferase (DGAT) increase during pregnancy toxemia in ewes [68]. Glycerophosphate acyltransferases 1 and glycerophosphate acyltransferases 2 account for 30-50% of total GPA activity in the liver [69]. Mice that lack GPAT1 exhibit reduced TG levels and increased BHBA concentrations in vivo [70,71]. Diacylglycerol acyltransferase catalyzes the partial esterification of fatty acyl groups to diacylglycerols to produce TGs [72]. Accordingly, triglyceride content was halved in mice lacking DGAT1 [73]. Moreover, *DGAT2*-deficient mice had less than 10% of the normal TG content, whereas *DGAT2* overexpression resulted in significantly increased TG content [74,75]. Thus, TG concentration relates to the level of TGS-related gene expression in the liver. In general, excessive accumulation of TGs in the liver causes fatty liver of ewes during pregnancy toxemia [20].

Lipoprotein lipase (LPL) is a critical factor in systemic lipid distribution and metabolism. LPL binds to the endothelial receptor glycosylphosphatidylinositol-anchored high-density lipoprotein-binding1 (GPIHBP1) and hydrolyzes TG-rich lipoproteins in capillaries, thereby providing fatty acids to heart and skeletal muscle [76]. A deficiency in LPL or GPIHBP1 impairs triglyceride hydrolysis and induces severe hypertriglyceridemia [77]. Angiopoietin-like proteins 3, 4, and 8 (ANGPTL3, -4, and -8) inhibit LPL activity. Angiopoietin-like protein 3 is expressed only in the liver, whereas ANGPTL4 and ANGPTL8 are enriched in both liver and adipose tissue [78]. Angiopoietin-like protein 4 may act as a local inhibitor of hepatic lipase and has also been proposed to function as an endocrine inhibitor of LPL in extrahepatic tissues [79]. Angiopoietin-like protein 4 influences circulating TGs by controlling local uptake of triglyceride-derived fatty acids in the tissue in which it is expressed [80]. In addition, overexpression of *ANGPTL4* decreased LPL activity, increased plasma TG levels, and triggered hypertriglyceridemia in a fasting mouse model [81], but the effect of ANGPTL4 was diminished during a long-term high-fat feeding program [82]. Angiopoietin-like protein 8 activated and promoted the secretion of ANGPTL3 in the liver by forming the ANGPTL3-8 complex, which inhibited LPL and increased plasma TG levels. The complex was more effective than ANGPTL3 alone in modulating TG concentrations in mice [83,84]. The expression of the liver *ANGPTL3* gene was downregulated significantly, and the *ANGPTL4* gene was upregulated due to increased BHBA and free fatty acid content in ewes with pregnancy toxemia [59]. In addition, ANGPTL3 controlled very low-density lipoprotein catabolism in a mouse model of hyperlipidemia [85]. The role of ANGPTL proteins in glucose and lipid metabolism and in cancer has been investigated intensively [86,87,88]. Certain ANGPTL proteins are promising pharmacological targets for hypertriglyceridemia [89], but the role of these proteins in ovine pregnancy toxemia requires further investigation.

## 5. Management Strategies for Pregnancy Toxemia

### 5.1. Diagnosis of Pregnancy Toxemia

#### 5.1.1. Histological Changes of Liver

Pregnancy toxemia in ewes is diagnosed based on pathological observations during necropsy, but the condition is not detected easily in the early stages of the disease. Extensive fat accumulation in the abdominal cavity and reduced rumen volume were observed during autopsy [21]. The liver of pregnancy toxemic ewes is enlarged, yellowish, and brittle, which may be due to progressive accumulation of TGs [68]. Meanwhile, the surface of the liver is locally hemorrhagic and degenerated. An increase in the nucleolus concentration and proliferation of glycogen granules in the cytoplasm were observed microscopically; hepatocytes appeared to be replaced by lipids with severe accompanying vacuolization [90,91]. Hematology, serum biochemistry, and ketone body level may help diagnose the disease (Table 2).

#### 5.1.2. Biochemical Parameters in the Blood

Pregnancy toxemia can be diagnosed by laboratory blood biochemical tests. Dairy sheep farmers must diagnose pregnancy toxemia early and accurately. It is usually easier to treat and prevent the diseases if they are diagnosed immediately and accurately.

Blood pH, pCO_2_ values, HCO_3_^-^ levels, and blood BE and K^+^ concentrations are significantly lower in dead goats than in surviving ones [94]. There was an explanation for hypokalemia, in part because the goats did not eat and so did not obtain enough dietary K. Pregnancy toxemia is often accompanied by acidosis, which may be associated with elevated unmeasured strong ions in goats [95]. Hypocalcemia can also occur with pregnancy toxemia [96]. The optimal threshold for calcium in clinical pregnancy toxemia is 7.13 mg/dL [93]. The only accurate way to differentiate between the two diseases at a farm level is to measure calcium and BHBA levels in the blood of affected animals [97]. Early postnatal biochemical parameters of pregnant ewes have significant value for the prognosis of lambs [98]. Fructosamine, creatinine, potassium, lactate dehydrogenase and malondialdehyde are the best prognostic indicators of pregnancy toxemia [93]. Lamb outcome is worse if lamb blood pH and blood base excess drop, whereas blood L-lactate and blood gas pCO_2_ levels rise [99].

Blood glucose in pregnant toxemic ewes is usually below 2 mMol/L [96]. Of the ewes with pregnant toxemia, 15.0% had hyperglycemia, 30.0% had normal blood glucose, and 55.0% had hypoglycemia [93]. As the primary metabolic fuel, glucose is essential to organ function, yet due to its strict homeostatic regulation, it is an insensitive energy indicator [100]. During the early stages, hypoglycemia is a diagnostic aid; however, as the disease progresses, ewes are recumbent and the blood glucose concentration increases [101].

Fructosamine and NEFA are the best diagnostic indicators of clinical and subclinical pregnancy toxemia [93]. Fructosamine shows the average blood glucose for the past 1–3 weeks [102]. The optimal thresholds for subclinical pregnancy toxemia and clinical pregnancy toxemia fructosamine were 1.005 mMol/L and 0.607 mMol/L, respectively [93]. When fructosamine levels are below 0.02 mMol/L, ewes have an almost 98% chance of dying, which makes fructosamine a good diagnostic indicator, as well as a good prognostic indicator [93]. The NEFA concentration can be increased above 0.4 mMol/L [96]. The optimal thresholds for NEFA in subclinical and clinical pregnancy toxemia are 0.390 mMol/L and 0.657 mMol/L, respectively [93]. The rise in NEFA levels can be attributed to hypoglycemia, which causes excessive mobilization of adipose tissue.

Pregnancy toxemia may be identified by increased urine ketone concentrations or increased serum BHBA concentrations. In previous studies in ewes, pregnancy toxemia during the last month of pregnancy was defined by a static threshold value [103]. Serum BHBA concentrations in normal pregnant ewes are typically <0.5 mMol/L, with clinical signs of pregnancy toxemia in ewes having BHBA concentrations of ≥3.0 mMol/L [104], >1.1 mMol/L [97]. In goats, subclinical pregnancy toxemia is defined as BHBA > 0.6 mMol/L [105]. In sheep, subclinical pregnancy toxemia is defined as 0.8–2.5 mMol/L [93]. Clinically normal ewes may develop ketonuria if plasma levels of BHBA reach 0.6 to 0.7 mMol/L [106]. Commercial qualitative test strips are used to measure ketone bodies in urine, and a positive result indicates ketonuria [107]. Ketonuria is a prodromal phase of pregnancy toxicity [104]. Measuring blood BHBA concentrations in the laboratory is the gold standard for diagnosing ketosis [108]. On-site measurements of BHBA and glucose concentrations for the diagnosis of pregnancy toxemia or ketosis are required due to the expense and time required to measure BHBA in the laboratory [109]. Handheld meters provide reliable, rapid, ewe-side early diagnosis of pregnancy toxemia in sheep [110], such as Nova Vet Meter^®^(Nova Biomedical, Waltham, MA), Precision Xceed^®^(Abbott, Abbott Diabetes Care Ltd., Oxon, UK). With the Nova Vet Meter^®^, ewe BHBA concentrations can be monitored within the range of 0.8 to 1.5 mMol/L through late gestation, but BHBA concentrations alone should not be used to differentiate subclinical ketosis from clinical ketosis [111]. The Precision Xceed^®^ is highly sensitive and specific, and has excellent test agreement for detection of animals with blood BHBA concentrations ≥0.8 mMol/L [109].

Increased LDH activity may be due to damage to the liver, skeletal muscle, and heart muscle in pregnancy toxemic ewes [93]. Increased AST and GGT activity indicates liver damage [90]. Pregnancy toxemia goats may experience myocardial damage, which significantly increases troponin and creatine kinase myocardial band values [112]. Thus, certain blood chemistry values may be useful in diagnosing ovine pregnancy toxemia (Table 2).

### 5.2. Treatment of Pregnancy Toxemia

Pregnancy toxemia has a long course and high mortality. Available treatments are not significantly effective. Pregnancy toxemia often leads to hypoglycemia, high blood and urine ketones, and acidosis. Therefore, treatment usually is based on glucose supplementation, liver protection, and detoxification. Prompt intervention in the early stages of the disease before irreversible nerve damage has occurred is key to the successful treatment of the condition [107]. Pregnancy toxemia-affected ewes should be treated without delay, and severe cases should be euthanized. It is possible to determine the extent of the risk in the flock by testing serum BHBA concentrations in late-gestation ewes. A general rule of thumb is to sample 10–20 animals in late gestation. The risk of the flock can be determined based on the value of BHBA: normal (low risk), 0–0.7 mmol/L; moderate underfeeding (moderate risk), 0.8–1.6 mmol/L; and severe underfeeding (high risk), 1.7–3.0 mmol/L [96].

The main treatment for the disease is glucose supplementation, which promotes enhanced glucose utilization at the tissue level and increases the turnover of ketone bodies and resolves ketoacidosis and electrolyte disturbances. Intravenous administration of a glucose solution in cases of severe hypoglycemia helps to reverse pathogenesis [97]. However, treatment with glucose in obese ewes with pregnancy toxemia may be harmful [92]. Supplemental propylene glycol and glycerol is a common treatment in the early stages of pregnancy toxemia (ambulatory, decreased appetite, and few CNS clinical signs) [113]. Administration of 100 mL/sheep/12 h of glycerol together with propylene glycol oral solution is perhaps the most effective treatment, as it normalizes biochemical parameters in a much shorter period of time [107]. A combination of 10 mg/kg of 2-methyl-2-phenoxy-propionic acid and 100 mL of propylene glycol oral can be effective in treating fatty liver and pregnancy toxemia [114]. A combination of intravenous lysine-glucagon (0.08 IU/kg body weight) and oral glucose solution (50 g) also may be beneficial [115]. Administration of flunixin meglumine at 2.5 mg/kg improves the survival rate of ewes and their lambs, although the mechanism is unknown [116].

Kang et al. [117] argued that the treatment of ketosis through targeted molecular pathways is an effective treatment for pregnancy toxemia. Supplemental ketone bodies reduced the proliferation of HMGCS2 knockdown cells, activated ketogenesis, and increased hepatic glucose production [61,118]. Momelolactone B and silibinin inhibited ketosis in vitro by inhibiting HMGCS2 [117,119] which suggests that these compounds may be promising therapeutic agents for pregnancy toxemia.

Induced parturition may also be advisable if the ewe is thin or overconditioned and cannot cope with the fetal demands [120]. Induced parturition or caesarean surgery were decided by the blood pH value [121]. When the pH of the PT goat at the first observation was below 7.15, a caesarean surgery was performed; when the pH was above 7.15, induction of parturition was the option utilized [94]. If the breeding date is known within 3 days, lambing can be induced in order to prevent preterm birth [122]. Breeders should follow the ewe closely in case of dystocia in artificial induction of parturition. The ewe and lambs may be saved if this course of action is followed soon. Ewes were injected with dexamethasone (16 or 25 mg doses) on either day 125, 135, or 141 of gestation to induce parturition for the purpose of reducing the uterine metabolic demand for energy [123]. Generally, lambing takes place between 36 and 60 h after induction [122]. Despite being recommended as the most effective method for inducing parturition in ewes, dexamethasone application seems an impractical method due to wide variations in lambing time [124]. Pregnancy toxemia is diagnosed during the final months of pregnancy, so it is impossible to predict when and how much dexamethasone will be applied [125]. In ewes with pregnancy toxemia, aglepristone (10 mg/kg) injections on days 140 and 141 of gestation could precisely control lambing timing without any side effects to the mother or lambs [126].

There is no effective treatment for late-stage pregnancy toxemia [9]. An already-comatose ewe should be euthanized, and the rest of the flock should be treated. If the breeders are determined to treat the ewe despite the poor prognosis, aggressive treatment should be directed against ketoacidosis and hypoglycemia. The fetuses should be checked for life (with real-time or Doppler ultrasounds) before treatment is started [127]. An emergency cesarean section may be considered if the fetuses are alive and within three days of their calculated due date [122]. If the fetus dies or is too early to survive a cesarean section, dexamethasone is used to induce early parturition in ewes. When the fetuses are considered dead, antibiotic therapy is recommended [122].

### 5.3. Prevention of Pregnancy Toxemia

In most instances, pregnancy toxemia can be prevented by balancing nutritional demands of the dam and increased requirements of the fetus during late gestation. Breeders may prevent pregnancy toxemia by optimizing the management of ewes. First, improving the breeding environment and avoiding ewe stress may be beneficial [128]. Second, dietary nutrition may be modified to prevent ewes from gaining excess fat or losing weight [129]. The feed for pregnant ewes should not only contain protein, vitamins, minerals, and other nutrients, but attention should also be paid to the reasonable combination of various nutrients, and the nutrients in the feed should be adjusted according to the weight of the pregnant ewes in a timely manner. The breeder can supply 250 g of concentrate per day during the second two months of pregnancy and increase it to 300–400 g/day two weeks before delivery [130]. Third, separate feeding of single and multiparous ewes may be helpful [131]. Fourth, monitoring of physiological and biochemical indicators of ewes throughout pregnancy may alert breeders to the development of pregnancy toxemia at an early stage [132]. In addition, monitoring of thyroid hormones, insulin, and glucose changes is an important tool to evaluate anabolic or catabolic adaptation in response to pregnancy and lactation in dairy cows [133]. We speculate that these methods also are applicable to negative energy balance and metabolic problems in ewes.

Medications that improve metabolism help reduced the prevalence of pregnancy toxemia. For example, a supplemental 10% crude glycerol in the diet [134] or intravenous 6 mL/ewe butyl phosphate and the synthetic vitamin B_12_ compound cyanocobalamin [135] improve the energy balance and metabolism of peripartum ewes. Nicotinamide, fumarolic acid, and per-rumen-protected choline improve ewe performance and lamb survival rates [136]. In addition, the injection of a single dose of 160 mg recombinant bovine somatotropin potentially has a preventive effect on pregnancy toxemia in sheep [137].

## 6. Conclusions

Sheep pregnancy toxemia is specific and complex, but the pathogenesis of the disease remains unclear. The condition is highly lethal and rarely reversible, and no effective methods of prevention or treatment are available currently. The study of disease regulatory mechanisms at the molecular level is important for the diagnosis and treatment of pregnancy toxemia, as well as for animal breeding and improvement. More research is required to uncover the mechanism of pregnancy toxemia in sheep, to achieve healthy reproduction in ewes, and to minimize economic losses in the sheep farming industry.

## Figures and Tables

**Table 1:** Fatty acid metabolism-related genes.

Description	Designation	Location
Fatty acid oxidation	Long-chain acyl-CoA synthase 1 (ASCL1)	Chromosome 3 (172306676..172309463)
	Long-chain acyl-CoA synthase 2 (ASCL2)	Chromosome 21 (45685760..45687628)
	Long-chain acyl-CoA dehydrogenase (ACADL)	Chromosome 2 (211860043..211904014, complement)
	Carnitine palmitoyltransferase 1B (CPT1B)	Chromosome 3 (225815614..225823875, complement)
	Carnitine palmitoyl transferase 1A (CPT1A)	Chromosome 21 (42553789..42613314, complement)
	Hydroxyacyl-CoA dehydrogenase (HADH)	Chromosome 6 (17565219..17608867, complement)
	Enolyl-CoA hydratase (ECH1)	Chromosome 14 (47913667..47922968, complement)
	Acetyl-CoA acyltransferase 1 (ACAA1)	Chromosome 23 (49746617..49779436, complement)
	Acetyl-CoA acyltransferase 2 (ACAA2)	Chromosome 19 (11608660..11633147, complement)
Acetyl coenzyme metabolism	3-Hydroxy-3-methylglutaryl-CoA (HMG-CoA)	
	Hydroxy methylglutaryl-CoA synthase 1 (HMGCS1)	Chromosome 16 (31543986..31566698)
	Hydroxy methylglutaryl-CoA synthase 2 (HMGCS2)	Chromosome 1 (97112935..97137264, complement)
	Hydroxy methylglutaryl-CoA lyase (HMGCL)	Chromosome 2 (242831761..242851036)
	Hydroxy methylglutaryl-CoA reductases (HMGCR)	Chromosome 7 (6749627..6771079)
Triglyceride synthesis	Glycerol kinase (GK)	Chromosome X (29849127..29924685)
	Glycerol-3-phosphate dehydrogenase 1 like (GPD1L)	Chromosome 19 (6617541..6675362)
	Glycerol-3-phosphate acyltransferase (GPA)	Chromosome 22 (32219904..32288784, complement)
	Diacylglycerol acyltransferase 1 (DGAT1)	Chromosome 9 (13560420..13569123)
	Diacylglycerol acyltransferase 2 (DGAT2)	Chromosome 15 (53943498..53976554)

**Table 2:** Changes in biochemical parameters during pregnancy toxemia.

Parameters	Pregnancy Toxemia	Reference Range	References
PH	↓	7.32–7.5	[92]
HCO_3_^−^ (mmol/L)	↓	20–29	[92]
BE (mmol/L)	↓	−5–+4	[92]
pCO_2_ (mmHg)	↓	38–45	[92]
Glucose (mg/dL)	↓	50–80	[93]
NEFA (mmol/L)	↑	<0.400	[93]
BHBA (mmol/L)	↑	<0.600	[94]
Fructosamine (mmol/L)	↓	1.25–1.36	[93]
Albumin (g/dL)	↓	2.4–3.0	[93]
Cholesterol (mg/dL)	↑	52–76	[93]
Triglyceride (mg/dL)	↑	12.22	[93]
AST (U/L)	↑	60–280	[93]
GGT (U/L)	↑	20–52	[93]
BUN (mmol/L)	↓	8–20	[93]
LDH (U/L)	↑	238–440	[93]
CK (U/L)	↑	8.1–12.9	[93]
Potassium (mEq/L)	↓	4.7–7.1	[93]
Calcium (mEq/L)	↓	11.5–12.8	[93]

BE, base excess; pCO_2_, partial pressure of carbon dioxide; BUN, blood urea nitrogen; BHBA, β-hydroxybutyrate; NEFA, non-esterified fatty acid; AST, aspartate aminotransferase; GGT, gamma-glutamyl transferase, LDH, lactate dehydrogenase; CK, creatinine kinase.
